# Echoes of the Month

**Published:** 1923-03

**Authors:** 


### ECHOES OF THE MONTH.

### CLEAN AND COOL MILK.

In a recent address to the Royal Sanitary Institute, Dr. Robertson, the Medical Officer of Health for Birmingham, after urging the necessity for educating the farmer in cleaner methods, said that it was of vital importance that the milk immediately it comes from the cow should be cooled down to (say) 50°Fahr. This is a matter of extreme difficulty in the case of many farms where the water supply is insufficient and cannot well be augmented. Where, however, such a condition of affairs exists, and indeed, in the case of many other farms, this difficulty could be got over by providing in the neighbourhood a brine cooling installation where the farmers' milk before being loaded into the train could be cooled. He also referred to another general principle as being essential to the production and transportation of clean milk, viz., that every article in which milk is received, from the farmer's milking cans to the railway churns and the bottles, should be not only cleaned but subsequently sterilised by steam. He found that at every little farm in America which he visited last summer a steamer was provided by the farmer to steam his milk cans and that every dairy had a machine which they called a churn washer, which perfectly washed, sterilised and dried the railway churns, and another similar apparatus which perfectly washed, sterilised and dried the delivery bottles. Dr. Robertson is a strong advocate of the supply of milk in bottles. The use of a dirty jug from the home can undo all the good that may be attributable to previous careful handling. In the past they have had very strong evidence in Birmingham that the home contamination is perhaps more deleterious than the dirt which the milk receives in the cowsheds or in course of transit on the railway. Bottled milk is free from the liability to convey disease; it keeps reasonably well and is a pleasant and easily-handled article of food.

### LIGHT AS A CAUSE OF DISEASE.

To " Cornhill " Miss Clara Boyle contributes an interesting article on " Light as a Cause of Disease, after Dr. Alois Czepa," from which we take the following extract : " Experiments were carried out with the so-called ' surviving ' frog's heart. The frog's heart is not very sensitive, and it is possible to keep it working normally for two or more days, though separated from its organism, when kept in a suitable solution containing nourishment and oxygen. Several frogs' hearts were kept in such a solution, some in the dark, some under rays of light, and no change could be observed in their regular beat. A solution containing hæmoporphyrin was then prepared, and several other frogs' hearts placed in this under observation. In the dark there was again no change, but under the influence of light the beating became irregular, and first the ventricles, then the auricles, came to a standstill. Such drugs as digitalis or atropine, which in other experiments soon showed a stimulant effect, remained here absolutely inactive. More careful examination disclosed that the muscle of the heart was in no way injured, but that the nerves of the heart had suffered severely. This result of the investigation coincided exactly with the experiments on living animals made sensitive to light, which always showed in the first instance a serious derangement of the nervous system, leading sooner or later to the death of the subject."

### CONDITIONAL NOTIFICATION.

Some novel recommendations are contained in the report of a Committee of the Board of Health in New Zealand, appointed to advise as to the best means of combating and preventing venereal disease in that Dominion. The outstanding recommendation of the report is the adoption of what is known as the system of conditional notification embodied in the West Australia Act. Under this plan the cases are notified by the doctor to the Health Department by number or symbol only. The name is not sent in unless the patient discontinues treatment before he is free from infection and refuses either to go to a clinic or to another doctor. In cases of those who " play the game," the name of the patient is kept confidential, and does not pass beyond the medical man attending him. It is also recommended that all proceedings taken under any Act having reference to venereal diseases should be held in private unless the defendant applies for a hearing in open court. The Committee suggest also that in any legislation which may be passed there should be a provision that every medical practitioner should give notice to the Director-General of Health, in the prescribed form, upon becoming aware that any person attended or treated by him is suffering from any venereal disease in a communicable form.

### IF SLEEPING SICKNESS CAME.

Writing in the " Empire Review " on " Cure of Sleeping Sickness," Dr. Andrew Balfour, C.M.G., says : " If sleeping sickness could establish itself here and begin to worry us and frighten us we would soon be up in arms against it. Our mental lethargy would vanish and we would quickly find methods of defeating it, for the latent talent of this country is wonderful, as the German found to his cost in the late war. As, however, there is no way of bringing home the importance of tropical diseases to the masses by violent measures, and as education regarding them operates slowly, the necessary effort must be made by the comparatively few who do understand. They must make the Government appreciate the situation and carry conviction also to our masters of commerce, for it is on trade that our whole fabric depends."

### "SHOCK-HEADED PETER" UP-TO-DATE.

The best mode of accustoming children to look after their own health is discussed in the *World's Health*—the monthly review of the League of Red Cross Societies—by Dr. Emmett Holt, the President of the Child Health Organisation of America. Dr. Holt starts from the assumption that before you can make a child enthusiastic about scrubbing its teeth, sleeping with the window well open, drinking milk when the elders drink tea, and so on, you must interest it in doing so. The children must be made to feel that health is a game with rules like other games, and the child's interest must be caught with something dramatic like Cho-Cho the Health Clown, the Jolly Jester, the Health Fairy, or some of the other dramatic characters developed by the Child Health Organisation. Or, again, children might be organised into " health clubs," of which the members wear a badge or uniform, and in which a regular programme of " health chores " is gone through. Children, Dr. Holt reminds us, love organisation and distinctive dress ; and a queer hat, we gather, may compensate even for organised tooth-brushing. Another suggestion is the cautionary tale, preferably in rhyme. Dr. Holt mentions as a useful example a verse which relates with commendable brevity that :—

> There was a boy in our town whose mother was not wise ;
> Coffee and tea he used to get and grew up undersize,
> But when he failed the football team because his size
> was small,
> He cut out both and took to milk, and grew up very tall.

This is by Mrs. Petersen, of whose Child Health Alphabet nearly 2½ million copies have been distributed in America.

### RADIO-ACTIVE WATER.

When Dr. Rosenfield recently visited the radium mines in Nevada, U.S.A., he found that the workers in the mines drink the water, which is strongly charged with radioactivity, and that their standard of health was above the normal, although their diet was the ordinary one. The President of an American Radium Institute, Dover, states that experiments on a number of patients have confirmed these observations. Aged people are said to have been restored to the mental and physical activities of healthy middle age, and rheumatism, diabetes, neuritis, and nervous disorders are reported to have been eliminated.

### WAR-TIME EXPERIENCE WITH CANNED FOODS.

" I never during the war saw a single case of food poisoning where the poisoning had arisen from the food being poisonous when it was in the unopened tin (it would have been brought to my notice if cases had occurred), and we had close on half a million troops in Mesopotamia—Indian, British and the Labour Corps, and accessory troops—and I can honestly say that not a single case was brought to my attention, and I think this was remarkable, especially in countries where the conditions were most favourable for food poisoning." So spoke Sir William Willcox, in an address to the Provision and Canned Goods Section of the London Chamber of Commerce. He afterwards heard of four cases, three in France and one in Port Said. The three in France were all due to contamination of the good wholesome tinned food by carriers in the process of making this food up into pies and stews. Two of them were due to the *bacillus aertrycke*, and one was due to the *bacillus enteritidis* of Gaertner. The Port Said epidemic was due to the contamination of wholesome tinned milk; it was due again to an infection from the carrier of the *bacillus aertrycke* that this epidemic of food poisoning occurred. Sir William adds that these remarkable facts have never before been brought to public notice.

### OCCUPATION AND HEALTH.

The Registrar-General has issued, as a supplement to his seventy-fifth Annual Report, Part IV., a report on the "Mortality of Men in Certain Occupations in the Years 1910, 1911, and 1912." During these years there was a "marked fall" as compared with 1900 to 1902. This amounted to 21 per cent. in the decennium for males aged 25 to 65. Among the most fatal occupations were tin miners, file makers, and costermongers and hawkers. All these stood 50 per cent. above the average. At the other end of the scale were "clergymen, ministers, &c." (55 per cent. below the average), gardeners and nurserymen (54 per cent. below), tallow, soap, and glue manufacturers (54 per cent. below).

### HOW X-RAYS LED TO RADIUM.

It was observed that the X-rays possessed the property of imparting a transient phosphorescence to glass, and this suggested the possibility that substances which could be made phosphorescent by the action of ordinary daylight might really be emitting rays which had the penetrating power and electrical properties of X-rays. For the most part this expectation was disappointed except in the case of certain compounds of uranium, on which Professor Becquerel carried out some experiments. He found that these compounds exceeded his expectations, inasmuch as they produced photographic and electrical effects without having been previously exposed to light. The active agent in them was discovered to be the uranium, which showed its activity in all its compounds. Then it was discovered that the ore residues from which the uranium had been extracted were even more active than the uranium itself, and by working with these residues Mme. Curie was able to isolate first polonium (so named in honour of her native country, Poland) and then radium.

### ENGLISH INDIFFERENCE TO DIRT.

"You English don't know how to handle food. I never saw such indifference to dirt." This, according to the "Westminster Gazette," is the opinion of a young American woman now living in London. "Look at your butchers' shops," she said, "and your fish shops, too—they are too disgusting for words—with the meat and fish exposed to the dirt and dust of the street, and your streets have plenty of both! In our New York shops we keep the meat and fish in ice-cooled, glass-cased storage cupboards, and when it is carried to our homes it is carefully and completely wrapped up in special waterproof paper. Then every American woman in her kitchen has a refrigerator in which all the perishable food is kept carefully shut up, in winter and summer, free from all danger of contamination and deterioration. Then take your milk. I never dreamed that such carelessness was possible. The way your milkmen handle the milk when they bring it round to your homes appals me. Such indifference to all the laws of health and cleanliness would not be tolerated in New York for five minutes; and it is not the law that would interfere, it would be a vigorous public opinion."

### GAS AS A NATIONAL ASSET.

In a syndicated article on Gas as a National Asset, Colonel Sir Arthur Holbrook, M.P., writes: "Precisely how vital and how necessary the gas undertakings of this country are was demonstrated during the war, and it can be truthfully said that no industry more than the gas industry helped to win the war. The products of gas works and gas ovens saved the Allies. This assistance took the form of the production of such necessary products as benzol and toluol for the manufacture of high explosives, dyes and motor spirit, sulphate of ammonia, creosote, tar, and carbolic." He points out also that if "England's dye industry and chemical trades are to be kept in existence, the gas industry must flourish. Out of smaller services rendered to the State many other industries have reached the peak of prosperity. Not so the British gas undertakings, which were seriously handicapped during the war, and have had tardy help since to recover their lost ground. But few people realise how closely coal distillation is bound up with food production. Vegetables cannot live without a proper supply of nitrogen, and before the war this country imported large quantities of nitrate of soda for this and other purposes. This supply was cut off during the war, so the gas works produced increased quantities of sulphate of ammonia, which serves the same useful purpose. Without this sulphate grass and green fields would yield but very poor results, and that would mean much less meat and corn for consumption."

### DISEASE AMONG ANATOLIAN REFUGEES.

The Rev. W. A. Wigram, D.D., Chaplain to the British Legation at Athens, sends to the *Times* a terrible description of the condition of the poor creatures who have taken refuge in Greece as the result of the recent Turkish victories. The Greek doctors, he says, whose standard of duty has hitherto not been low, are beginning to resent the claims made upon them, and refuse to visit refugees. "Dear Madam, consider how dirty they are, and the price of a suit of clothes in these days," said one to an English lady who begged him to come to the place where she had been toiling. "Our paying patients do not like us to go among the typhus and smallpox and then come to them," said another. Hence appalling things are beginning to happen in remoter districts. There was one place in Epirus (let us admit that it only befell after two Greek doctors had died in their duty) where all the victims of typhus and smallpox were herded together in one room and left there to recover or die. No mediæval lazar-house could have been worse than this—and we must add that the big room in question was just over the only grocer's shop of the village. The American Red Cross heard of the state of things and took drastic measures for its betterment, so that this is now past; but it may befall again elsewhere.

### PASTEUR'S DISCOVERIES ANTICIPATED.

A volume has recently been discovered on a bookstall on the Paris Quays which suggests that the discoveries associated with Pasteur had been anticipated by a Frenchman named Boile living in the age of Louis XV. and of his consulting physician, Astruc. According to the Paris correspondent of the *Morning Post*, the great Astruc has pilloried in a vehement Latin treatise this bold investigator, accusing him of asserting that "all diseases were caused by small animals enclosed in the blood, that each disease is due to different animals, that these mischievous animals have each of them in particular other animals for their enemy which pursue them and destroy them," and so on. Astruc has no hesitation in describing this student of microbes and bacteria as a "cunning charlatan," condemns as a fraud "the microscope with which Boile claimed that he could prove to the naked eye all that he asserted," and confesses that "I do not know what he (Boile) expected to gain by his trickeries, but I do know that he had the prudence to avoid by flight the punishment he deserved." Apparently Boile had not the courage to face the Court doctors, and having made his discovery thought it best to disappear.

### WHY HAVE A HEALTH WEEK?

In connection with Health Week, October 8-14 last, arrangements were made with the approval of the Education Committee of the London County Council, by which the head teachers in the Central Higher Grade and elementary schools were invited to organise essay competitions in their schools on the subject, "Why have a Health Week?" for which prizes were offered. The head teacher selected the best essay in each senior department, and 83 essays were sent for consideration of the examiners appointed by the Health Week Committee. The examiners awarded 50 prizes, and the committee express their appreciation of the careful and systematic instruction in the essentials of hygiene and healthy living which is being given in the schools.

